# Correlation analysis between preoperative systemic immune inflammation index and prognosis of patients after radical gastric cancer surgery: based on propensity score matching method

**DOI:** 10.1186/s12957-021-02457-2

**Published:** 2022-01-03

**Authors:** Xu Zhaojun, Chen Xiaobin, An Juan, Yuan Jiaqi, Jiang Shuyun, Liu Tao, Cai Baojia, Wang Cheng, Ma Xiaoming

**Affiliations:** 1 Graduate School, Qinghai University, Xining, 810001 China; 2 Department of Gastrointestinal Surgery, Qinghai University Affiliated Hospital, Xining, 810001 China; 3 Department of General Surgery, 900th Hospital of Joint Logistics Support Force, Fuzhou City, 350001 Fujian Province China; 4 Department of Basic Medical Sciences, Qinghai University Medical College, No.251 of Ningda Road, Xining City, 810016 Qinghai Province China; 5 State Key Laboratory of Plateau Ecology and Agriculture, Qinghai University, No.251, Ningda Road, Xining City, 810016 Qinghai Province China

**Keywords:** Gastric carcinoma, Systemic immune inflammation index, Prognosis, Overall survival, Propensity score matching method

## Abstract

**Background:**

To explore the correlation between the preoperative systemic immune inflammation index (SII) and the prognosis of patients with gastric carcinoma (GC).

**Methods:**

The clinical data of 771 GC patients surgically treated in the Department of Gastrointestinal Surgery, Qinghai University Affiliated Hospital from June 2010 to June 2015 were retrospectively analyzed, and their preoperative SII was calculated. The optimal cut-off value of preoperative SII was determined using the receiver operating characteristic (ROC) curve, the confounding factors between the two groups were eliminated using the propensity score matching (PSM) method, and the correlation between preoperative SII and clinicopathological characteristics was assessed by chi-square test. Moreover, the overall survival was calculated using Kaplan-Meier method, the survival curve was plotted, and log-rank test was performed for the significance analysis between the curves. Univariate and multivariate analyses were also conducted using the Cox proportional hazards model.

**Results:**

It was determined by the ROC curve that the optimal cut-off value of preoperative SII was 489.52, based on which 771 GC patients were divided into high SII (H-SII) group and low SII (L-SII) group, followed by PSM in the two groups. The results of Kaplan-Meier analysis showed that before and after PSM, the postoperative 1-, 3-, and 5-year survival rates in L-SII group were superior to those in H-SII group, and the overall survival rate had a statistically significant difference between the two groups (*P* < 0.05). Before PSM, preoperative SII [hazard ratio (HR) = 2.707, 95% confidence interval (CI) 2.074-3.533, *P* < 0.001] was an independent risk factor for the prognosis of GC patients. After 1:1 PSM, preoperative SII (HR = 2.669, 95%CI 1.881–3.788, *P* < 0.001) was still an independent risk factor for the prognosis of GC patients.

**Conclusions:**

Preoperative SII is an independent risk factor for the prognosis of GC patients. The increase in preoperative SII in peripheral blood indicates a worse prognosis.

## Introduction

Gastric carcinoma (GC) is a common digestive tract malignancy. In 2018, there were up to 782,000 deaths of GC, making it the third cause of death in malignancies [1]. China is a GC-prone country, and the new cases and deaths account for 44.1% and 49.9%, respectively, of the total globally, with an age-standardized 5-year survival rate of 27.4%. At the same time, GC patients have insidious early symptoms and the therapeutic effect is poor, leading to a poor prognosis [2]. Therefore, researchers have been constantly exploring the new simple, economical and accurate prognostic evaluation index similar to TNM stage currently, so as to better guide the clinical treatment [3].

According to related studies, both inflammatory response and immune response are closely related to the occurrence and development of tumors [4, 5]. Tumor-induced inflammatory response can cause corresponding changes in the blood neutrophil (NE), lymphocyte (LY), and platelet (PLT) counts [6–8]. On this basis, some studies have tried to discover new potential biomarkers related to the prognosis. For example, the preoperative systemic immune inflammation index (SII) calculated based on NE, LY, and PLT is correlated with the prognosis of breast cancer, liver cancer and pancreatic cancer [9–12]. However, there are currently few studies on the correlation between SII and prognosis of GC. In the present study, therefore, the value of SII for the prognosis of GC patients was explored using propensity score matching (PSM), so as to provide references for clinical treatment.

## Methods

This study was agreed by the Institutional Research Ethics Board of Qinghai University Affiliated Hospital and obtain the informed consent of all subjects themselves or their families. All methods were performed in accordance with the Declaration of Helsinki, and this study did not involve human or animal tissue or blood samples, and all patients signed the written informed consent before surgery.

### General data

The clinical data of GC patients surgically treated in the Department of Gastrointestinal Surgery, Qinghai University Affiliated Hospital from June 2010 to June 2015 were retrospectively analyzed. The treatment methods included distal gastrectomy (DG), proximal gastrectomy (PG) and total gastrectomy (TG). Inclusion criteria were as follows: (1) patients pathologically diagnosed with gastric mucosal adenocarcinoma after operation, (2) those without undergoing radiotherapy, chemotherapy and biotherapy before operation, (3) those without acute/chronic inflammation before operation, (4) those undergoing standard D2 gastrectomy, (5) those without other severe concomitant diseases and with good organ function, and (6) those without medical contraindications that seriously affect anesthesia and operation. Exclusion criteria were as follows: (1) patients with missing medical data or lost to follow-up, (2) those who refused to undergo operation, (3) those complicated with blood system diseases or other tumors before operation, (4) those with severe uncontrolled recurrent infections, or other severe uncontrolled concomitant diseases, or (5) those who require immunosuppressive therapy due to organ transplantation. The pathological staging was based on the 8th edition UICC/AJCC staging criteria [13], and the pathological diagnosis and classification of GC were based on the *Japanese Gastric Cancer Treatment Guidelines (5th edition)* [14].

### Analysis methods

SII was calculated using the blood routine test results first time after admission (SII = PLT × NE/LY). According to the optimal cut-off value of SII, the patients were grouped, and the correlation between SII and clinicopathological factors of patients was analyzed. The patients were also divided into non-elderly group (< 60 years old) and elderly group (≥ 60 years old). The correlation between SII and patients’ age and its influence on prognosis were analyzed.

### PSM

This was a retrospective study, the data were not strictly randomized and there were many potential influencing factors for SII in clinic, so various confounding factors might be distributed in GC patients with different SII. To eliminate the influence of confounding factors on the prognosis, 1:1 PSM was performed for patients in high SII (H-SII) group and low SII (L-SII) group. Other covariates had no statistically significant difference (*P* > 0.05).

### Follow-up

All patients were regularly followed up after operation every 3–6 months by outpatient clinic, message, telephone, e-mail, and network communication tools. Overall survival (OS) was defined as the duration from definite diagnosis to death or end of follow-up (February 2021 or patients’ death).

### Statistical analysis

SPSS 26.0, R 3.6.1, and GraphPad Prism 8.0 were used for statistical analysis. Enumeration data were expressed as *n* (%), and *χ*^2^ test was performed for intergroup comparison. The optimal cut-off value of SII was determined using the receiver operating characteristic (ROC) curve. The confounding factors in data were eliminated using PSM, and the matching precision was set to 0.02. OS was calculated using Kaplan-Meier method, the survival curve was plotted, and log-rank test was performed for the difference between groups. Univariate and multivariate analyses were also conducted using the Cox proportional hazards model. The hazard ratio (HR) and the corresponding 95% confidence interval (95% CI) were calculated. *P* < 0.05 was considered to be statistically significant.

## Results

### General data

**Fig. 1:**
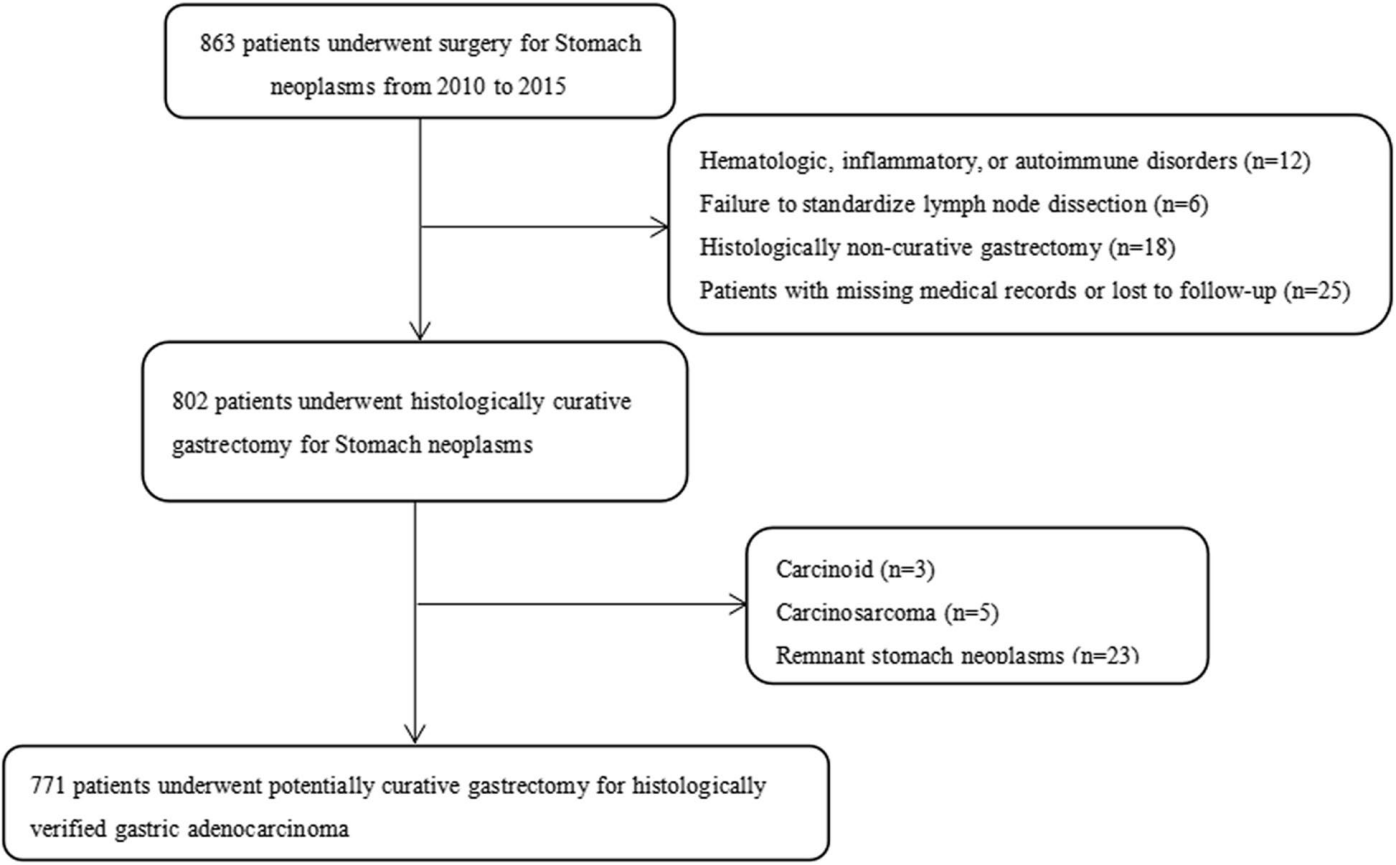
Study flow diagram

A total of 771 patients met the screening criteria, including 165 females (21.40%) and 606 males (78.60%), with a median age of 59.30 years old (Fig. 1). All patients were pathologically diagnosed with gastric mucosal adenocarcinoma after operation, and none of them died of postoperative complications. Among all patients, 297 cases (38.52%) underwent DG, 250 cases (32.43%) underwent PG, and 224 cases (29.05%) underwent TG. Standard D2 lymph node dissection was conducted for all patients. All of the 771 patients were followed up until 6 February 2021, with a median follow-up time of 46 months (95% CI 43.986–48.014).

### Optimal cut-off value of SII

**Fig. 2:**
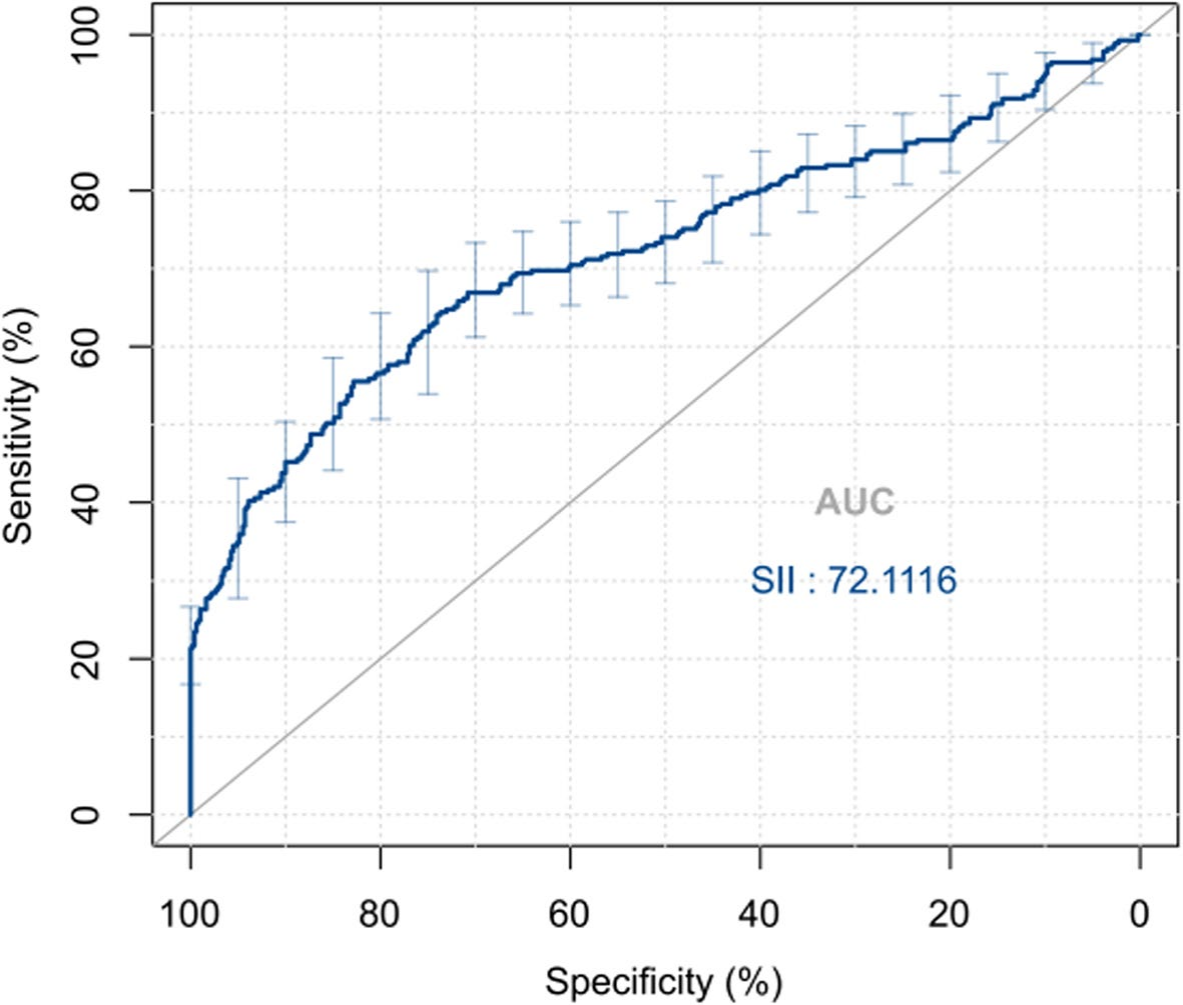
ROC curves were plotted to determine the optimal cut-off value of SII

The ROC curve of SII was plotted, and its AUC and Youden index were 0.721 (95%CI 0.681–0.761) and 0.384, respectively. The corresponding optimal cut-off value was 489.52, and the evaluation sensitivity and specificity were 55.5% and 82.9%, respectively (Fig. 2). Based on the cut-off value, 771 patients were divided into L-SII group (SII ≤ 489.52, *n* = 531) and H-SII group (SII > 489.52, *n* = 240).

### Correlation between SII and clinicopathological factors of GC patients before and after PSM

Before matching, SII was related to the maximum diameter of tumor, histological type, serum albumin (ALb), white blood cell (WBC), carbohydrate antigen 125 (CA125), and TNM stage (*P* < 0.05), but not related to the age, gender, smoking, drinking, carcinoembryonic antigen (CEA), CA199, and operation method (*P* > 0.05). PSM was performed in L-SII group and H-SII group, and a total of 354 patients were eligible, including 177 cases in H-SII group and 177 cases in L-SII group. The above differences were evenly distributed after matching (*P* > 0.05) (Table 1).

**Table 1:** Correlations between SII and clinicopathological factors of GC patients before and after PSM

Parameters	Before PSM				After PSM			
	H-SII (*n* = 240)	L-SII (*n* = 531)	***χ***^2^	***P***	H-SII (*n* = 177)	L-SII (*n* = 177)	***χ***^2^	***P***
**Age (years)**			3.415	0.065			0.011	0.915
≤ 60	129(53.75%)	323(60.83%)			100(56.50%)	99(55.93%)		
> 60	111(46.25%)	208(39.17%)			77(43.50%)	78(44.07%)		
**Gender**			0.097	0.756			0.000	1.000
Male	187(77.92%)	419(78.91%)			138(77.97%)	138(77.97%)		
Female	53(22.08%)	112(21.09%)			39(22.03%)	39(22.03%)		
**Smoking**			0.234	0.628			0.011	0.915
Yes	113(47.08%)	260(48.96%)			84(47.46%)	83(46.89%)		
No	127(52.92%)	271(51.04%)			93(52.54%)	94(53.11%)		
**Drinking**			1.246	0.264			1.457	0.227
Yes	91(37.92%)	224(42.18%)			72(40.68%)	61(34.46%)		
No	149(62.08%)	307(57.82%)			105(59.32%)	116(65.54%)		
**Tumor size (mm)**			11.357	< 0.001			0.232	0.630
≤ 50	168(70.00%)	429(80.79%)			128(72.32%)	132(74.58%)		
> 50	72(30.00%)	102(19.21%)			49(27.68%)	45(25.42%)		
**Differentiation**			4.188	0.041			0.000	1.000
Well and moderately	73(30.42%)	202(38.04%)			61(34.46%)	61(34.46%)		
Poorly	167(69.58%)	329(61.96%)			116(65.54%)	116(65.54%)		
**Alb (g/L)**			33.695	< 0.001			0.000	1.000
≤ 39.85	183(76.25%)	288(54.24%)			121(68.36%)	121(68.36%)		
> 39.85	57(23.75%)	243(45.76%)			56(31.64%)	56(31.64%)		
**WBC(W, × 10**^9^ cells/L)			107.173	< 0.001			0.113	0.737
≤ 6.525	119(49.58%)	451(84.93%)			118(66.67%)	115(64.97%)		
> 6.525	121(50.41%)	80(15.07%)			59(33.33%)	62(35.03%)		
**CEA**			0.542	0.462			0.554	0.457
≤ 2.17 ug/ml	128(53.33%)	268(50.47%)			89(50.28%)	82(46.33%)		
> 2.17 ug/ml	112(46.67%)	263(49.53%)			88(49.72%)	95(53.67%)		
**CA199**			0.835	0.361			0.0137	0.711
≤ 21 u/mL	179(74.58%)	412(77.59%)			135(76.27%)	132(74.58%)		
> 21 u/mL	61(25.42%)	119(22.41%)			42(23.73%)	45(25.42%)		
**CA125**			6.671	0.010			1.532	0.216
≤ 14.6 u/ml	145(60.42%)	371(69.87%)			112(63.28%)	123(69.49%)		
> 14.6 u/ml	95(39.58%)	160(30.13%)			65(36.72%)	54(30.51%)		
**Type of surgery**			5.572	0.062			4.974	0.084
DG	80(33.33%)	217(40.87%)			57(32.21%)	39(22.04%)		
PG	78(32.50%)	172(32.39%)			59(33.33%)	63(35.59%)		
TG	82(34.17%)	142(26.74%)			61(34.46%)	75(42.37%)		
**TNM stage**			11.509	0.003			2.425	0.298
I	38(15.83%)	137(25.80%)			34(16.95%)	35(19.77%)		
II	43(17.92%)	105(19.78%)			31(17.51%)	40(22.60%)		
III	159(66.25%)	289(54.42%)			116(65.54%)	102(57.63%)		

*H-SII* high systemic immune inflammatory group, *L-SII* low systemic immune inflammatory group, *ALb* albumin, *WBC* white blood cell, *DG* distal gastectomy, *PG* proximal gastrectomy, *TG* total gastrectomy

### Correlation between SII and OS of GC patients before and after PSM

**Fig. 3:**
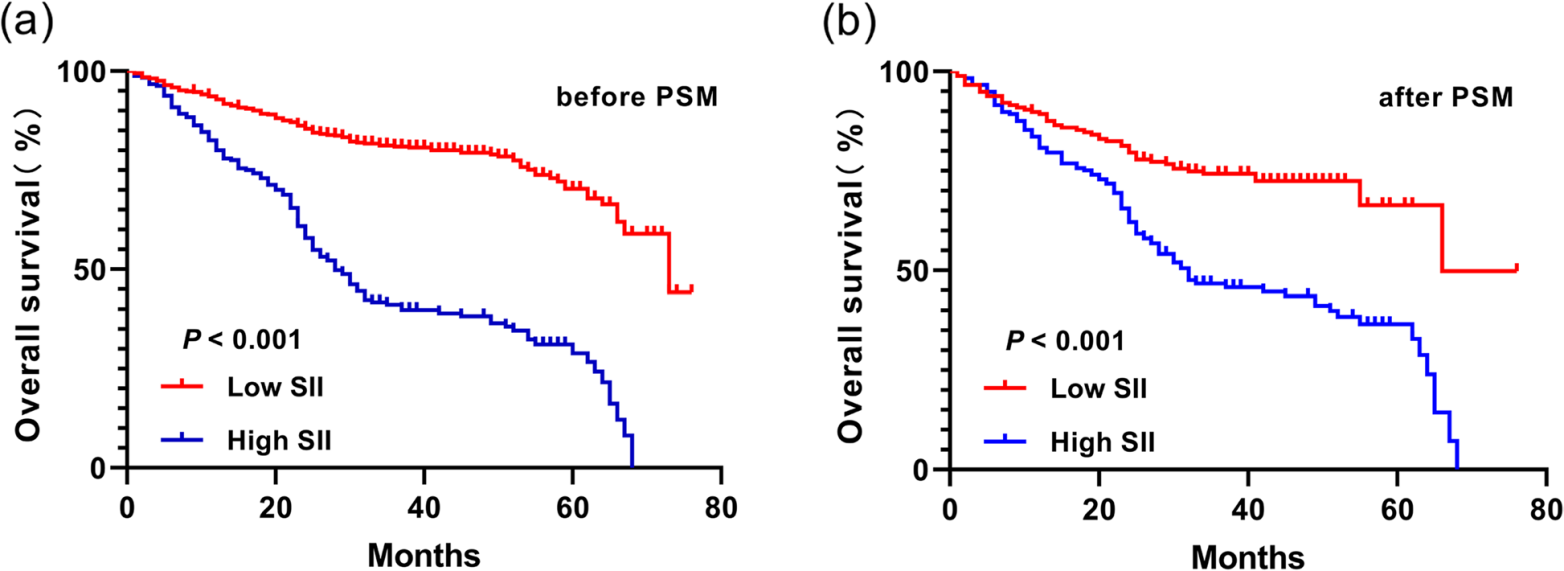
Postoperative OS curve in the two groups before and after PSM. **a** Before PSM, the 5-year OS rate in L-SII group was significantly higher than that in H-SII group (67.9% vs. 28.9%) (*P* < 0.001). **b** After PSM, the 5-year OS rate in L-SII group was significantly higher than that in H-SII group (60.4% vs. 32.9%) (*P* < 0.001)

The median survival time was 73 months (95%CI 65.172–80.828) in L-SII group and 28 months (95%CI 24.646–31.354) in H-SII group. The 1-, 3-, and 5-year survival rates were 92.8%, 81.0%, and 67.9%, respectively, in L-SII group, and 80.0%, 39.8%, and 28.9%, respectively, in H-SII group. It can be seen that the survival rate in L-SII group was superior to that in H-SII group, and the OS rate had a statistically significant difference between the two groups (*P* < 0.001). After PSM, the 1-, 3-, and 5-year survival rates were 89.3%, 74.3%, and 60.4%, respectively, in L-SII group, and 80.8%, 45.8%, and 32.9%, respectively, in H-SII group. The mean survival time was 56.771 months (95%CI 51.613–61.929) in L-SII group. In H-SII group, the mean survival time was 38.443 months (95%CI 34.792–42.093), and the median survival time was 32 months (95%CI 22.446–41.554). It can be seen that the survival rate in L-SII group was significantly better than that in H-SII group, and the OS rate had a statistically significant difference between the two groups (*P* < 0.001) (Fig. 3).

### Influencing factors for patient’s survival before and after PSM

**Fig. 4:**
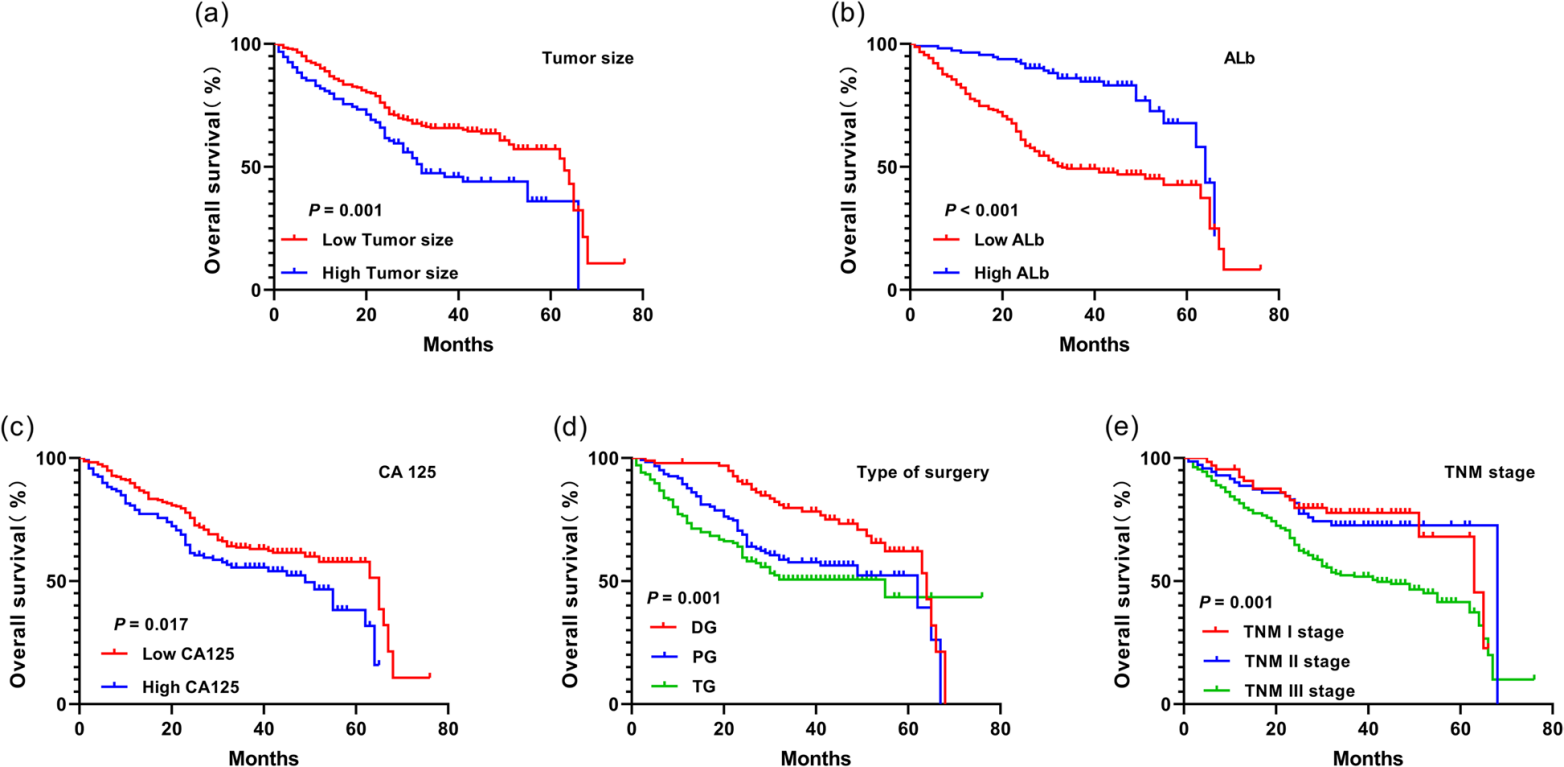
Survival curves under different risk factors for the prognosis after PSM. **a** different tumor sizes (H-SII group vs. L-SII group). **b** Different ALb levels (H-SII group vs. L-SII group). **c** Different CA125 levels (H-SII group vs. L-SII group). **d** Different operation methods (DG vs. PG vs. TG). **e** Different TNM stages (stage I vs. stage II vs. stage III)

Before PSM, the results of univariate analysis revealed that age, maximum diameter of tumor, histological type, SII, ALb, CEA, CA199, CA125, WBC count, operation method, and TNM stage were all influencing factors for the prognosis of GC patients (*P* < 0.05). The results of multivariate analysis showed that age (HR = 1.334, 95%CI 1.045–1.704, *P* = 0.021), histological type (HR = 0.741, 95%CI 0.560–0.980, *P* = 0.036), SII (HR = 2.707, 95%CI 2.074–3.533, *P* < 0.001), ALb (HR = 0.385, 95%CI 0.283–0.525, *P* < 0.001), CEA (HR = 1.285, 95%CI 1.007–1.640, *P* = 0.044), CA125 (HR = 1.370, 95%CI 1.069–1.755, *P* = 0.013), WBC (HR = 1.361, 95%CI 1.047–1.770, *P* = 0.021), operation method, and TNM stage were all independent risk factors for the prognosis of GC patients (Table 2). After PSM, the results of univariate analysis manifested that age, maximum diameter of tumor, SII, ALb, CA125, operation method, and TNM stage were risk factors for the prognosis of GC patients. According to multivariate analysis, the maximum diameter of tumor (HR = 1.493, 95%CI 1.043–2.136, *P* = 0.029), SII (HR = 2.669, 95%CI 1.881–3.788, *P* < 0.001), ALb (HR = 0.286, 95%CI 0.183–0.447, *P* < 0.001), CA125 (HR = 1.402, 95%CI 1.002–1.961, *P* = 0.048), operation method, and TNM stage were independent risk factors for the prognosis of GC patients (Table 3). Besides, the correlation between statistically significant independent risk factors after PSM (*P* < 0.05) (except SII) and prognosis of GC patients was analyzed by Kaplan-Meier method. It was found that the larger maximum diameter of tumor, lower level of ALb, higher level of CA125 and higher TNM stage indicated a worse prognosis. Besides, the prognosis was better among patients undergoing DG than that among patients treated with PG and TG (Fig. 4).

**Table 2:** Risk factors for the prognosis of GC patients before PSM

Parameters	Univariate analysis		Multivariate analysis	
	HR (95% CI)	***P***	HR (95% CI)	***P***
**Age, years**(≤ 60 vs. > 60)	1.531(1.209–1.938)	< 0.001	1.334(1.045–1.704)	0.021
**Gender** (Male vs. Female)	0.792(0.584–1.072)	0.131		
**Smoking** (Yes vs. No)	0.913(0.722–1.155)	0.448		
**Drinking** (Yes vs. No)	0.981(0.774–1.244)	0.876		
**Tumor size, mm**(≤ 5 vs. >5)	1.619(1.253–2.091)	< 0.001	1.182(0.903–1.547)	0.223
**Differentiation** (poorly vs. well and moderately)	0.605(0.462–0.794)	< 0.001	0.741(0.560–0.980)	0.036
**SII**(> 489.52 vs. ≤ 489.52)	3.919(3.087–4.977)	< 0.001	2.707(2.074–3.533)	< 0.001
**Alb, g/L**(≤ 39.85 vs. > 39.85)	0.298(0.220–0.403)	< 0.001	0.385(0.283–0.525)	< 0.001
**CEA, ug/ml**(≤ 2.17 ug/ml vs. > 2.17 ug/ml)	1.329(1.050–1.680)	0.018	1.285(1.007–1.640)	0.044
**CA199, u/ml**(≤ 21 u/mL vs. > 21 u/mL)	1.560(1.208–2.013)	0.001	1.184(0.905–1.550)	0.218
**CA125, u/ml**(≤ 14.6 u/ml vs. > 14.6 u/ml)	1.658(1.307–2.104)	< 0.001	1.370(1.069–1.755)	0.013
**WBC**,10^9^/L(≤ 6.525 vs. > 6.525)	2.158(1.698–2.742)	< 0.001	1.361(1.047–1.770)	0.021
**Type of surgery**		< 0.001		< 0.001
DG	1(reference)		1(reference)	
PG	1.781(1.325–2.395)		1.728(1.279–2.337)	
TG	2.331(1.736–3.129)		1.878(1.386–2.545)	
**TNM stage**		< 0.001		0.016
I	1(reference)		1(reference)	
II	1.574(1.004–2.466)		1.248(0.788–1.978)	
III	2.385(1.665–3.416)		1.654(1.136–2.408)	

*HR* hazard ratio, *CI* confidence interval, *SII* systemic immune inflammatory, *ALb* albumin, *WBC* white blood cell, *DG* distal gastectomy, *PG* proximal gastrectomy, *TG* total gastrectomy

**Table 3:** Risk factors for the prognosis of GC patients after PSM

Parameters	Univariate analysis		Multutivariate analysis	
	HR (95% CI)	***P***	HR (95% CI)	***P***
**Age,y**(≤60 VS. >60)	1.544(1.121-2.126)	0.008	1.356(0.973-1.889)	0.072
Gender (male VS. female)	0.958(0.649-1.415)	0.83		
Smoking (yes VS. no)	1.023(0.744-1.407)	0.887		
Drinking (yes VS. no)	1.098(0.790-1.527)	0.577		
**Tumor size,mm**(≤5 VS. >5)	1.729(1.237-2.417)	0.001	1.493(1.043-2.136)	0.029
**Differentiation** (Poorly VS. Well and Moderately)	0.733(0.514-1.046)	0.087		
**SII**(>489.52 VS. ≤489.52)	2.472(1.757-3.480)	<0.001	2.669(1.881-3.788)	<0.001
**Alb,g/L**(≤39.85 VS. >39.85)	0.306(0.198-0.474)	<0.001	0.286(0.183-0.447)	<0.001
**CEA,ug/ml**(≤2.17ug/ml VS. >2.17ug/ml)	1.380(0.999-1.906)	0.051		
**CA199,u/ml**(≤21u/mL VS. >21u/mL)	1.415(0.996-2.011)	0.053		
**CA125,u/ml**(≤14.6u/mll VS. >14.6u/ml)	1.479(1.067-2.050)	0.019	1.402(1.002-1.961)	0.048
**WBC**^,109/L^(≤6.525 VS. >6.525)	0.921(0.659-1.288)	0.631		
**Type of surgery**		0.001		0.001
DG	1(reference)		1(reference)	
PG	1.803(1.159-2.805)		2.061(1.314-3.232)	
TG	2.230(1.451-3.428)		2.324(1.490-3.623)	
**TNM stage**		<0.001		0.002
I	1(reference)		1(reference)	
II	1.803(1.159-2.805)		1.120(0.579-2.167)	
III	2.230(1.451-3.428)		2.123(1.257-3.584)	

*HR* hazard ratio, *CI* confidence interval, *SII* Systemic immune inflammatory, *ALb* Albumin, *WBC* White blood cell, *DG* Distal gastectomy, *PG* Proximal gastrectomy, *TG* Total gastrectom

### Clinicopathological characteristics of age-stratified patients after PSM

After PSM, 199 non-elderly patients (56.21%) and 155 elderly patients (43.79%) were obtained for analysis. It was proved that SII was related to the TNM stage of non-elderly patients (*P* < 0.05) (Table 4).

**Table 4:** Age-stratified PSM

Parameters	Non-elderly patients(*n* = 199)				Elderly patients(*n* = 155)			
	H-SII(*n* = 100)	L-SII(*n* = 99)	***χ***^2^	***P***	H-SII(*n* = 77)	L-SII(*n* = 78)	***χ***^2^	***P***
**Gender**			0.106	0.744			0.102	0.749
Male	80(80.00%)	81(81.82%)			58(75.32%)	57(73.08%)		
Female	20(20.00%)	18(18.18%)			19(24.68%)	21(26.92%)		
**Smoking**			0.245	0.621			0.516	0.472
Yes	47(47.00%)	50(50.51%)			37(48.05%)	33(42.31%)		
No	53(53.00%)	49(49.49%)			40(51.95%)	45(57.69%)		
**Drinking**			0.445	0.505			1.145	0.285
Yes	42(42.00%)	37(37.37%)			30(38.96%)	24(31.17%)		
No	58(58.00%)	62(62.63%)			47(61.04%)	54(70.13%)		
**Tumor size (mm)**			0.834	0.361			0.102	0.749
≤ 50	70(70.00%)	75(75.76%)			58(75.32%)	57(73.08%)		
> 50	30(30.00%)	24(24.24%)			19(24.68%)	21(26.92%)		
**Differentiation**			0.065	0.799			0.073	0.787
Well and moderately	33(33.00%)	68(68.69%)			49(63.64%)	48(61.54%)		
poorly	67(67.00%)	31(31.31%)			28(36.36%)	30(38.46%)		
**Albumin (g/L)**			0.056	0.813			0.113	0.737
≤ 39.85	61(61.00%)	62(62.63%)			60(77.92%)	59(75.64%)		
> 39.85	39(39.00%)	37(37.37%)			17(22.08%)	19(24.36%)		
**WBC**, 10^9^/L			0.411	0.521			0.052	0.820
≤ 6.525	65(65.00%)	60(60.61%)			53(68.83%)	55(70.51%)		
> 6.525	35(35.00%)	39(39.39%)			24(31.17%)	23(29.49%)		
**CEA**			0.047	0.828			0.78	0.377
≤ 7 ug/ml	48(48.00%)	46(46.46%)			41(53.25%)	36(46.15%)		
> 7 ug/ml	52(52.00%)	53(53.54%)			36(46.75%)	42(53.85%)		
**CA199**			0.085	0.771			0.773	0.379
≤ 21 u/mL	75(75.00%)	76(76.77%)			60(77.92%)	56(71.79%)		
> 21 u/mL	25(25.00%)	23(23.23%)			17(22.08%)	22(28.21%)		
**CA125**			3.336	0.068			0.01	0.921
≤ 14.6 u/ml	65(65.00%)	76(76.77%)			47(61.04%)	47(60.26%)		
> 14.6 u/ml	35(35.00%)	23(23.23%)			30(38.96%)	31(39.74%)		
**Type of surgery**			2.206	0.332			3.085	0.214
DG	36(36.00%)	26(26.26%)			21(27.27%)	13(16.67%)		
PG	32(32.00%)	36(36.37%)			27(35.07%)	27(34.62%)		
TG	32(32.00%)	37(37.37%)			29(37.66%)	38(48.71%)		
**TNM stage**			14.683	0.001			4.102	0.129
I	14(14.00%)	18(18.18%)			16(20.78%)	17(21.79%)		
II	9(9.00%)	28(28.28%)			22(28.57%)	12(15.38%)		
III	77(77.00%)	53(53.54%)			39(50.65%)	49(62.83%)		

*H-SII* high systemic immune inflammatory group, *L-SII* low systemic immune inflammatory group, *ALb* albumin, *WBC* white blood cell, *DG* distal gastectomy, *PG* proximal gastrectomy, *TG* total gastrectomy

### Cox regression analysis on influencing factors for the prognosis of non-elderly patients

The results of univariate analysis showed that the maximum diameter of tumor, SII, ALb, CA125, and TNM stage were risk factors for the prognosis of the 199 non-elderly patients with GC. The results of multivariate analysis showed that SII (HR = 3.383, 95%CI 1.961–5.838, *P* < 0.001) and ALb (HR = 0.400, 95%CI 0.238–0.674, *P* = 0.001) were independent risk factors for the prognosis of non-elderly patients (Table 5).

**Table 5:** Univariate and multivariate analyses on OS of non-elderly patients

Parameters	Univariate analysis		Multivariate analysis	
	HR (95% CI)	***P***	HR (95% CI)	***P***
**Gender** (male vs. female)	0.834(0.459–1.516)	0.552		
**Smoking** (yes vs. no)	1.042(0.663–1.639)	0.858		
**Drinking** (yes vs. no)	1.090(0.688–1.726)	0.714		
**Tumor size, mm**(≤ 5 vs. >5)	1.878(1.177–2.995)	0.008	1.452(0.893–2.359)	0.133
**Differentiation** (poorly vs. well and moderately)	0.926(0.561–1.528)	0.764		
**SII**(> 489.52 vs. ≤ 489.52)	3.970(2.334–6.751)	< 0.001	3.383(1.961–5.838)	< 0.001
**Albumin, g/L**(≤ 39.85 vs. > 39.85)	0.462(0.277–0.769)	0.003	0.400(0.238–0.674)	0.001
**CEA,ug/ml**(≤ 2.17 ug/ml vs. > 2.17 ug/ml)	1.208(0.768–1.901)	0.413		
**CA199,u/ml**(≤ 21 u/mL vs. > 21 u/mL)	1.110(0.660–1.867)	0.693		
**CA125,u/ml**(≤ 14.6 u/ml vs. > 14.6 u/ml)	1.783(1.124–2.830)	0.014	1.592(0.992–2.554)	0.054
**WBC**, 10^9^/L(≤ 6.525 vs. > 6.525)	0.981(0.616–1.561)	0.934		
**Type of surgery**		0.104		
DG	1(reference)			
PG	1.801(1.039–3.123)			
TG	1.296(0.723–2.324)			
**TNM stage**		0.004		0.090
I	1(reference)		1(reference)	
II	1.644(0.549–4.920)		2.111(0.686–6.493)	
III	3.539(1.423–8.799)		2.776(1.091–7.063)	

*HR* hazard ratio, *CI* confidence interval, *SII* systemic immune inflammatory, *ALb* albumin, *WBC* white blood cell, *DG* distal gastectomy, *PG* proximal gastrectomy, *TG* total gastrectomy

### Cox regression analysis on influencing factors for the prognosis of elderly patients

The results of univariate analysis showed that the histological type, SII, ALb, CEA, CA199, operation method, and TNM stage were risk factors for the prognosis of the 155 elderly patients with GC. It was confirmed by multivariate analysis that SII (HR = 2.372, 95%CI 1.444–3.896, *P* = 0.001), ALb (HR = 0.164, 95%CI 0.059–0.457, *P* = 0.001), and operation method were independent risk factors for the prognosis of elderly patients (Table 6).

**Table 6:** Univariate and multivariate analyses on OS of elderly patients

Parameters	Univariate analysis		Multivariate analysis	
	HR (95% CI)	***P***	HR (95% CI)	***P***
**Gender** (male vs. female)	0.976(0.574–1.661)	0.928		
**Smoking** (yes vs. no)	0.960^(^0.610–1.513^)^	0.861		
**Drinking** (yes vs. no)	1.024^(^0.635–1.652^)^	0.922		
**Tumor size, mm**(≤ 5 vs. > 5)	1.610(0.993–2.611)	0.053		
**Differentiation** (poorly VS. well and moderately)	0.532(0.321–0.881)	0.014	0.689(0.410–1.158)	0.160
**SII**(> 489.52 vs. ≤ 489.52)	1.612(1.012–2.567)	0.044	2.372(1.444–3.896)	0.001
**Alb, g/L**(≤ 39.85 vs. > 39.85)	0.132(0.048–0.361)	< 0.001	0.164(0.059–0.457)	0.001
**CEA, ug/ml**(≤ 2.17 ug/ml vs. > 2.17 ug/ml)	1.704(1.069–2.715)	0.025	1.530(0.930–2.517)	0.094
**CA199, u/ml**(≤ 21 u/mL vs. > 21 u/mL)	1.848(1.138–3.002)	0.013	1.457(0.877–2.421)	1.146
**CA125, u/ml**(≤ 14.6 u/ml vs. > 14.6 u/ml)	1.142(0.718–1.818)	0.574		
**WBC**, 10^9^/L(≤ 6.525 vs. > 6.525)	0.940(0.574–1.539)	0.806		
**Type of surgery**		< 0.001		0.002
DG	1(reference)		1(reference)	
PG	1.893(0.875–4.098)		1.548(0.709–3.381)	
TG	4.004(1.937–8.279)		3.174(1.490–6.760)	
**TNM stage**		0.020		0.090
I	1(reference)		1(reference)	
II	0.902(0.397–2.049)		0.766(0.328–1.786)	
III	1.914(1.020–3.590)		1.539(0.798–2.967)	

*HR* hazard ratio, *CI* confidence interval, *SII* systemic immune inflammatory, *ALb* albumin, *WBC* white blood cell, *DG* distal gastectomy, *PG* proximal gastrectomy, *TG* total gastrectomy

### Postoperative survival rate of non-elderly and elderly patients

**Fig. 5:**
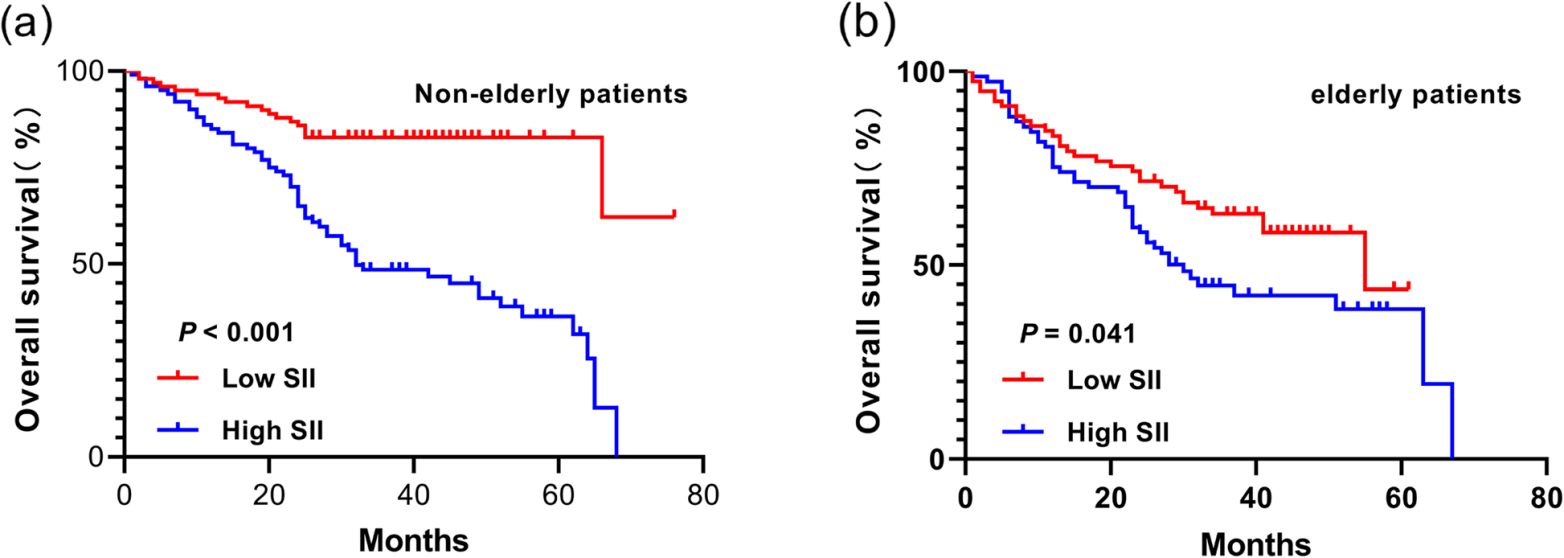
Correlation between the prognosis of non-elderly and elderly patients and SII after PSM. **a** The 5-year OS rate in L-SII group was significantly higher than that in H-SII group among the non-elderly patients (62.1% vs. 31.9%) (*P* < 0.001). **b** The 5-year OS rate in L-SII group was significantly higher than that in H-SII group among the elderly patients (43.8% vs. 19.4%) (*P* = 0.041)

Among the non-elderly patients, the 1-, 3-, and 5-year survival rates were 92.9%, 82.8% and 62.1%, respectively, in L-SII group, and 85.0%, 47.6%, and 31.9%, respectively, in H-SII group. The survival rate of GC patients in L-SII group was better than that in H-SII group, and the OS rate had a statistically significant difference between the two groups (*P* < 0.001). Among the elderly patients, the 1-, 3-, and 5-year survival rates were 83.3%, 63.2%, and 43.8%, respectively, in L-SII group, and 75.3%, 42.2%, and 19.4%, respectively, in H-SII group. The survival rate of GC patients in L-SII group was better than that in H-SII group, and the OS rate had a statistically significant difference between the two groups (*P* = 0.041) (Fig. 5).

## Discussion

In recent years, related studies have demonstrated that the inflammatory response (tissue necrosis, activation of immune response, secretion of cytokines) plays an important role in the occurrence, development and metastasis of tumors [15, 16]. In clinical practice, medical staff often use various methods such as enteral nutrition support and combined ERAS to adjust the immunity of cancer patients to improve the prognosis of patients [17, 18]. In the early stage, the inflammatory response is induced and the anti-tumor immune response is inhibited, thereby promoting growth of tumor cells. With the growth of tumor, the inflammatory response is further enhanced. To eliminate inflammation, the recruitment of immune cells to tumor tissues is reduced, resulting in immune escape of tumor cells. In the late stage, the body’s immunity is almost lost, and the inflammatory response is further worsened, thus facilitating the tumor progression [19]. In recent years, researches on the prognosis of gastric cancer patients have been intensified. Follistatin-like 1 (FSTL1), miR-23b, ETS1, and TCF4 have all been reported to be prognostic biomarkers for gastric cancer patients, mainly involved in tumor progression and tumor immunity [20, 21]. Related literature has reported that neutrophil-to-lymphocyte ratio (NLR) and platelet-to-lymphocyte ratio (PLR) are significantly related to the prognosis of gastric cancer patients. High levels of NLR and PLR before surgery are the prognostic factors for poor OS in gastric cancer patients [22, 23]. SII, a new inflammatory index integrating the NE, LY and PLT counts [24], can reflect the balance status between tumor immunity and inflammatory response more comprehensively than platelet-to-lymphocyte ratio (PLR), neutrophil-to-lymphocyte ratio (NLR) and monocyte-to-lymphocyte ratio (MLR), and better predict the prognosis of cancer patients [24–26]. Inoue Hiroki et al. [27] pointed out that GC patients with SII ≥ 395 (HR = 2.95, 95%CI 1.07–4.07, *p* = 0.001) had poor OS; the high SII group had a higher peritoneal recurrence rate than the low SII group (*p* = 0.028). In this study, based on previous studies, the propensity score matching (PSM) method was used to explore the value of SII on the prognosis of patients with gastric cancer, and to provide references for clinical treatment.

In the present study, the optimal cut-off value of SII was determined using the ROC curve, based on which the patients were divided into H-SII group and low L-SII group. In terms of the correlation between SII and the clinicopathological characteristics of patients, SII was related to the maximum diameter of tumor, histological type, ALb, WBC, CA125 and TNM stage (P<0.05). In addition, the survival time of GC patients in H-SII group was significantly shortened, and the 5-year survival rate was significantly lower than that in L-SII group. Inoue [27] *et al.* retrospectively analyzed the clinical data of 447 GC patients treated with gastrectomy and found that the 5-year survival rate is 80.0% and 92.7%, respectively, in H-SII group and L-SII group, suggesting that the SII level is related to the prognosis of GC patients, consistent with the results of this study. Han BL [28] also showed that SII is closely related to the prognosis of GC patients, and high SII indicates a poor prognosis of GC patients. Its mechanism can be explained by the functions of NE, PLT and LY. In the case of tumor invasion, the inflammatory response can promote the increase in NE and the secretion of NO, reactive oxygen species, arginase and other active substances, which can regulate the activity of NK cells and LY, thereby facilitating tumor growth and metastasis [29, 30]. In addition, the PLT count rises in inflammation, and PLT promotes tumor angiogenesis via releasing vascular endothelial growth factor and platelet-derived growth factor [31], creating favorable conditions for tumor growth. Moreover, PLT can form colonizing tumor thrombi with tumor cells, thus promoting the further invasion of tumor cells into the body. On the contrary, LY can effectively suppress the occurrence and development of tumors, and induce the death of tumor cells. The long-term inflammatory stimulus will lead to decline in LY subsets, thereby causing immune dysfunction, and the decrease in LY count can raise the risk of immune escape of tumor cells [32–34]. Therefore, SII can reflect the relation between systemic inflammatory response and body’s immunity during tumor progression. The higher the SII is, the more severe the inflammatory response and immunosuppression in GC patients will be.

In this study, it was confirmed by univariate analysis before and after PSM that age, maximum diameter of tumor, SII, ALb, CA125, operation method and TNM stage were risk factors for the prognosis of GC patients. Before PSM, the results of multivariate analysis showed that age, histological type, SII, ALb, CEA, CA125, WBC, operation method, and TNM stage were independent risk factors for the prognosis of GC patients. After PSM, the results of multivariate analysis revealed that the maximum diameter of tumor, SII, ALb, CA125, operation method, and TNM stage were independent risk factors for the prognosis of GC patients. Then the statistically significant factors after PSM (except SII) were analyzed. It was found that the larger maximum diameter of tumor, lower level of ALb, higher level of CA125, and higher TNM stage indicated a worse prognosis. The prognosis was better among patients undergoing DG than that among patients treated with PG and TG. Wang et al. [35] found in the study on the correlation between SII and the prognosis of GC patients that SII and TNM stage are independent risk factors for the prognosis of GC patients, consistent with the results of this paper. As the main component of plasma protein, ALb can reflect the nutritional status of patients, and it has also been widely used as an antidote and transporter in predicting the survival status and disease progression of cancer patients [36, 37]. CA125 is mainly present in epithelial tissues and serum of patients, and its expression level in the serum of GC patients is higher than that in normal people, which is positively correlated with the TNM stage and negatively correlated with the prognosis of GC patients, consistent with the results of this study. This indicates that CA125 can be used to assess the severity of GC [38]. In addition, the multivariate analysis before and after PSM demonstrated that no positive result was obtained in age after PSM, but other scholars argued that there is a correlation between age and the prognosis of GC patients [39, 40]. The reasons why age was not an independent risk factor for the prognosis of GC patients in this study are as follows: PSM was used in this study to validate the impact of SII on the prognosis on the basis of excluding other confounding factors, which stressed the effect of SII on the prognosis, thus affecting the prognostic assessment by other indexes to a certain extent. Besides, it is possibly because the sample size was small after PSM and this study itself had limitations, rather than simply considering age unrelated to the prognosis of patients. Therefore, the sample size remains to be further expanded in the future. To further explore the correlation between age and the prognosis of GC patients, age was further subjected to stratification analysis to exclude the value deviation in a certain stage caused by the small sample size or the bias of age stratification in this study. It is reported in the literature that elderly patients are aged ≥ 60 years old [41, 42]. In this study, the median age of 354 patients was 59.0 years old after PSM. Therefore, patients aged ≥ 60 years old were defined as elderly patients in this study. The results revealed that the mean SII of elderly patients (495.73) was significantly higher than that of non-elderly patients (443.21), suggesting the correlation between SII and patient’s age. In addition, the risk factors for the prognosis of GC patients were analyzed under age stratification. It was found that the high SII was correlated with the poor prognosis of elderly patients with GC, and the 5-year OS rate of elderly patients was lower than that of non-elderly patients in H-SII group (31.9% vs. 62.1%). The specific mechanism remains to be further confirmed by a large number of prospective studies.

There were certain limitations in this study. First, this was a small-sample retrospective study on GC patients in high-altitude areas. To better control the bias, PSM was used for data analysis, but all the impact of covariates on the outcome failed to be fully eliminated. Second, this was a single-center study, so the conclusion cannot fully represent the characteristics of other study centers and populations, limiting its popularization. Therefore, the preliminary results need to be validated by larger-sample randomized controlled studies in the future, so as to offer more convincing theoretical support to the existing conclusion.

In conclusion, SII, that remains simple, universal, non-invasive, cheap, and reproducible, is expected to be an index for assessing the prognosis of GC patients.

## Data Availability

The datasets generated during and/or analyzed during the current study are available from the corresponding author on reasonable request.

## Abbreviations

SII: Systemic immune inflammation index
GC: Gastric carcinoma
ROC: Receiver operating characteristic
PSM: Propensity score matching
H-SII: High SII
L-SII: Low SII
HR: Hazard ratio
CI: Confidence interval
NE: Neutrophil
LY: Lymphocyte
PLT: Platelet
DG: Distal gastrectomy
PG: Proximal gastrectomy
TG: Total gastrectomy
OS: Overall survival
ALb: Albumin
WBC: White blood cell
CA125: Carbohydrate antigen 125
CEA: Carcinoembryonic antigen
PLR: Platelet-to-lymphocyte ratio
NLR: Neutrophil-to-lymphocyte ratio
MLR: Monocyte-to-lymphocyte ratio

## References

[1] Bray F (2018). Global cancer statistics 2018: GLOBOCAN estimates of incidence and mortality worldwide for 36 cancers in 185 countries. CA Cancer J Clin.

[2] Gao K, Jun W (2019). National trend of gastric cancer mortality in China (2003–2015): a population-based study. Cancer Commun (Lond).

[3] Park L, HoJae (2015). Overview of gastrointestinal cancer prevention in Asia. Asia Pacific Microwave Conference.

[4] Prete AD, Allavena P, Santoro G (2011). Molecular pathways in cancer-related inflammation. Biochem Med.

[5] Candido J, Hagemann T. Cancer-related inflammation. J Clin Immunol. 2013;33 Suppl 1:S79–84. 10.1007/s10875-012-9847-0. 10.1007/s10875-012-9847-0 23225204

[6] Mizunuma M, Yokoyama Y, Futagami M, Aoki M, Takai Y, Mizunuma H. The pretreatment neutrophil-tolymphocyte ratio predicts therapeutic response to radiation therapy and concurrent chemoradiation therapy in uterine cervical cancer. Int J Clin Oncol. 2015;20(5):989–96. 10.1007/s10147-015-0807-6. 10.1007/s10147-015-0807-6 25736530

[7] Labelle M, Begum S, Hynes RO (2011). Direct signaling between platelets and carcinoma cells induces an epithelial-mesenchymal-like transition and promotes metastasis. Cancer Cell.

[8] Lizhen Z (2016). A new prognostic score based on the systemic inflammatory response in patients with inoperable non-small-cell lung cancer. OncoTargets Ther.

[9] Gao YB, Guo W, Cai SH (2019). Systemic immune-inflammation index (SII) is useful to predict survival outcomes in patients with surgically resected esophageal squamous cell carcinoma. J Cancer.

[10] Wang P, Yue WS, Li WY (2019). Systemic immune-inflammation index and ultrasonographic classification of breast imaging-reporting and data system predict outcomes of triple-negative breast carcinoma. Cancer Manag Res.

[11] Hu B, Yang XR, Xu Y (2014). Systemic immune-inflammation index predicts prognosis of patients after curative resection for hepatocellular carcinoma. Clin Cancer Res.

[12] Aziz MH, Sideras K, Aziz NA (2019). The systemic-immune-inflammation index independently predicts survival and recurrence in resectable pancreatic carcinoma and its prognostic value depends on bilirubin levels: a retrospective multicenter cohort study. Ann Surg.

[13] Fei S, Ziyu L, Lianhai Z, Shuangxi L, Yongning J, Rulin M, Xue K, Zhemin L, Gao X, Wang Y, Chao Y, Shen L, Jiafu J (2017). The Union for International Cancer Control (UICC) and the American Joint Committee on Cancer (AJCC) gastric cancer TNM staging system (8^th^ edition) explanation and elaboration. Chin J Pract Surg.

[14] Xiang H (2019). Changes of Japanese "Guidelines" and New Trends of Gastric Cancer Treatment. Chin J Pract Surg.

[15] Grivennikov SI, Greten FR, Karin M (2010). Immunity, inflammation, and carcinoma. Cell.

[16] Singh R, Mishra MK, Aggarwal H (2017). Inflammation, immunity, and cancer. Mediat Inflamm.

[17] Xin F, Mzee SAS, Botwe G, He H, Zhiyu S, Gong C, Said ST, Jixing C (2019). Short-term evaluation of immune levels and nutritional values of EN versus PN in gastric cancer: a systematic review and a meta-analysis. World J Surg Oncol.

[18] Zhou J, Lin S, Sun S, Zheng C, Wang J, He Q (2021). Effect of single-incision laparoscopic distal gastrectomy guided by ERAS and the influence on immune function. World J Surg Oncol.

[19] Huang B (2012). Regulation of immune response and inflammation in tumor microenvironment. Chin J Cancer Biother.

[20] Mei D, Qi Y, Xia Y, Ma J, Hu H, Ai J, Chen L, Wu N, Liao D (2021). Microarray profile analysis identifies ETS1 as potential biomarker regulated by miR-23b and modulates TCF4 in gastric cancer. World J Surg Oncol.

[21] Li L, Huang S, Yao Y, Chen J, Li J, Xiang X, Deng J, Xiong J (2020). Follistatin-like 1 (FSTL1) is a prognostic biomarker and correlated with immune cell infiltration in gastric cancer. World J Surg Oncol.

[22] Kim EY, Song KY (2020). The preoperative and the postoperative neutrophil-to-lymphocyte ratios both predict prognosis in gastric cancer patients. World J Surg Oncol.

[23] Zhang X, Zhao W, Yu Y, Qi X, Song L, Zhang C, Li G, Yang L (2020). Clinicopathological and prognostic significance of platelet-lymphocyte ratio (PLR) in gastric cancer: an updated meta-analysis. World J Surg Oncol.

[24] Hongyuan F (2018). Systemic immune-inflammation index (SII) is useful to predict survival outcomes in patients after liver transplantation for hepatocellular carcinoma within Hangzhou Criteria. Cell Physiol Biochem Pharmacol.

[25] Diem S, Schmid S, Krapf M, Flatz L, Born D, Jochum W, Templeton AJ, Früh M (2017). Neutrophil-to-lymphocyte ratio (NLR) and platelet-to-lymphocyte ratio (PLR) as prognostic markers in patients with non-small cell lung cancer (NSCLC) treated with nivolumab. Lung Cancer.

[26] Trifan G, Testai FD (2020). Systemic Immune-Inflammation (SII) index predicts poor outcome after spontaneous supratentorial intracerebral hemorrhage. J Stroke Cerebrovasc Dis.

[27] Inoue H, Kosuga T, Kubota T, Konishi H, Shiozaki A, Okamoto K, Fujiwara H, Otsuji E (2021). Significance of a preoperative systemic immune-inflammation index as a predictor of postoperative survival outcomes in gastric cancer. World J Surg Oncol.

[28] Han BL, Wang YM, Xue YW (2019). Effect of preoperative systemic immune-inflammation index on the prognosis of patients with gastric cancer. Chin J Gen Surg.

[29] Hu XX, He YF, Luo HQ, Chen WJ, Ke LH, Yan Y, Wu SS, Niu JY, Li HM, Xu HJ, Hu B (2019). The relationship between peripheral blood NLR, PLR and clinical prognosis of small cell esophageal cancer. Chin Clin Oncol.

[30] Teramukai S, Kitano T, Kishida Y (2009). Pretreatment neutrophil count as an independent prognostic factor in advanced non-small-cell lung cancer: An analysis of Japan Multinational Trial Organisation LC00-03. Eur J Cancer.

[31] Brill A, Dashevsky O, Rivo J, Gozal Y, Varon D (2005). Platelet-derived microparticles induce angiogenesis and stimulate post-ischemic revascularization. Cardiovasc Res.

[32] Hong H (2017). Prognostic value of preoperative NLR, dNLR, PLR and CRP in surgical renal cell carcinoma patients. World J Urol.

[33] Ozmen S (2017). Neutrophil-lymphocyte ratio (NLR) and platelet-lymphocyte ratio (PLR) may be superior to C-reactive protein (CRP) for predicting the occurrence of differentiated thyroid cancer. Endocr Regul.

[34] Song W, Bai Y, Zhu J, Zeng F, Yang C, Hu B, Sun M, Li C, Peng S, Chen M, Sun X (2021). A novel prognostic model based on epithelial-mesenchymal transition-related genes predicts patient survival in gastric cancer. World J Surg Oncol.

[35] Qingshan W, Dayong Z (2019). The prognostic value of systemic immune-inflammation index (SII) in patients after radical operation for carcinoma of stomach in gastric cancer. J Gastrointestinal Oncol.

[36] Abe A (2020). Correlation between prognostic nutritional index and occlusal status in gastric cancer. Oral Dis.

[37] Mantzorou M, Koutelidakis A, Theocharis S (2017). Clinical value of nutritional status in cancer: what is its impact and how it affects disease progression and prognosis?. Nutr Cancer.

[38] Chao H (2019). Clinical significance of serum CA125, CA19-9, CA72-4, and fibrinogen-to-lymphocyte ratio in gastric cancer with peritoneal dissemination. Front Oncol.

[39] Zou XN, Wan X, Dai Z, Yang GH (2012). Epidemiological characteristics of cancer in elderly chinese. ISRN Oncol.

[40] Yan S, Li B, Bai Z-Z, Jun-Qi W, Xie D-W, Ma Y-C, Ma X-X, Zhao J-H, Guo X-J (2014). Clinical epidemiology of gastric cancer in Hehuang valley of China:A 10-year epidemiological study of gastric cancer. World J Gastroenterol.

[41] Yang LD, Wang XD, Li Q, Liang YH, Chen Y, Wu MJ, Xu F (2015). The safety in more than 60-year-old gastric cancer patients treated by fast-track surgery. J Modern Oncol.

[42] Xu PW, Zhang H (2015). Clinical manifestations, pathological features and prognosis of elderly patients with gastric cancer. J Nanjing Med Univ (Natural Science).

